# Could Ultrasound Be Used as a Triage Tool in Diagnosing Fractures in Children? A Literature Review

**DOI:** 10.3390/healthcare10050823

**Published:** 2022-04-29

**Authors:** Roxana Iacob, Emil Robert Stoicescu, Simona Cerbu, Daniela Iacob, Elena Amaricai, Liliana Catan, Oana Belei, Emil Radu Iacob

**Affiliations:** 1 Department of Radiology and Medical Imaging, ‘Victor Babes’ University of Medicine and Pharmacy Timisoara, 300041 Timisoara, Romania; roxana.iacob@umft.ro (R.I.); stoicescu.emil@umft.ro (E.R.S.); cerbusimona@yahoo.com (S.C.); 2 Research Center for Pharmaco-Toxicological Evaluations, ‘Victor Babes’ University of Medicine and Pharmacy Timisoara, 300041 Timisoara, Romania; 3 Department of Neonatology, ‘Victor Babes’ University of Medicine and Pharmacy Timisoara, 300041 Timisoara, Romania; 4 Department of Rehabilitation, Physical Medicine and Rheumatology, Research Center for Assessment of Human Motion, Functionality and Disability, “Victor Babes” University of Medicine and Pharmacy Timisoara, 300041 Timisoara, Romania; amaricai.elena@umft.ro (E.A.); catan.liliana@umft.ro (L.C.); 5 Department of Pediatrics, First Pediatric Clinic, Disturbances of Growth and Development on Children Research Center, Victor Babes University of Medicine and Pharmacy, 30041 Timisoara, Romania; belei.oana@umft.ro; 6 Department of Pediatric Surgery, ‘Victor Babes’ University of Medicine and Pharmacy, 300041 Timisoara, Romania; radueiacob@umft.ro

**Keywords:** imaging, ultrasound, fractures, children, musculoskeletal ultrasound

## Abstract

Fracture is one of the most frequent causes of emergency department visits in children, conventional radiography being the standard imaging tool used for following procedures and treatment. This imagistic method is irradiating and harmful, especially for children due to their high cell division rate. For this reason, we searched the literature to see if musculoskeletal ultrasound is a good alternative for diagnostic and follow-up regarding fractures in the pediatric population. After searching the databases using MeSH terms and manual filters, 24 articles that compare X-ray and ultrasound regarding their specificity and sensitivity in diagnosing fractures were included in this study. In the majority of the studied articles, the specificity and sensitivity of ultrasound are around 90–100%, and with high PPVs (positive predictive values) and NPVs (negative predictive values). Although it cannot replace conventional radiography, it is a great complementary tool in fracture diagnosis, having a sensitivity of nearly 100% when combined with clinical suspicion of fracture, compared with X-ray.

## 1. Introduction

In children, fractures are some of the most frequent emergencies and are considered a consequence of normal active upbringing [1,2,3,4,5]. The occurrence rate of fractures in children is 9.47/1000, with boys being more likely to be injured than girls [1,3]. Sports injuries are the most common causes of fracture, whereas the anatomic region that is the most affected during childhood is the distal forearm, followed by the lower limb, with an age peak between 9–14 years [1,2,6,7,8,9]. As studies show, children with previous bone injuries are more likely to sustain a new fracture in the future [2,10].

The fracture treatment aims to regain complete function of the injured part, as well as to obtain a good cosmetic result [11,12]. Fractures can affect the child’s growth and development due to malalignment of the bone, length overgrowth, compartment syndrome, or neurovascular complications [2,13,14]. Due to these consequences, the correct management of fractures plays a very important role in the recovery of the bone [15,16]. Initial evaluation of the injury must include the history of the trauma, physical examination of the affected segment, including neurovascular and pulse assessment (before fracture reduction), as well as plain radiographs that must include the joints above and below the fracture site [17,18,19,20,21,22].

Although conventional radiography is the gold standard for fracture diagnosis and evaluation, it is well-known that this method could cause long-term complications, such as malignancy development, especially in children, whose cellular division ratio is higher than in adults [23,24,25,26]. Additionally, taking into consideration the fact that because of the COVID-19 pandemic, the radiology departments are overcrowded, finding another way to evaluate fractures is needed [27,28]. The opposite way stands ultrasonography, which is radiation-free, could be used in evolution and the child can be accompanied by a parent during the procedure, is considered a more friendly examination tool [29,30]. For these reasons, musculoskeletal ultrasound could be a valuable diagnostic tool for fractures, for evaluating the callus development, an important step that is required in follow-up treatment [31,32].

The objective of this study is to compare fracture ultrasonography with conventional radiography and to find the accuracy of echography—sensitivity and specificity—in order to use this non-invasive method in the near future for diagnosing and monitoring pediatric fractures. Moreover, considering the pandemic situation, a method of screening that can be used not only by the radiologist, but also by emergency physicians or orthopedic surgeons with ultrasound skills could be very helpful.

## 2. Materials and Methods

Articles included in this literature review were chosen in accordance with PRISMA (Preferred Reporting Items for Systematic Reviews and Meta-Analyses) guidelines. We conducted the literature search in October 2021 using PubMed database, and Cochrane, searching for studies published between 2011 to 2021, resulting in a 10-year period. The following MeSH Therms were used in PubMed database: ((“fractures, bone/diagnostic imaging” [MeSH Terms]) AND (“ultrasonography” [MeSH Terms])) AND (“radiography” [MeSH Terms]).

The predetermined list of exclusion criteria included:

(1) Non-English study;
(2) Articles published more than 10 years ago;
(3) Studies that were not applied to human subjects;
(4) Studies that were conducted on patients older than 18 years old, taking into account that the majority of the authors work in the pediatric field;
(5) Studies that did not use ultrasonography as an alternative method in the diagnosis or follow-up of fractures;
(6) No comparison of ultrasonography with radiography;
(7) Studies were conducted on other bones rather than the limbs.

The database with the selected articles was achieved with Microsoft Excel. The table had the following columns: article title, authors, year of publication, journal, type of publication, keywords, and PubMed article link.

Based on the anatomical region described in each study, the main ideas, and the conclusions each author had, we focused, when possible, on seven directions:

1. US compared with X-ray in the diagnosis of long bones fractures.
2. US compared with X-ray in the diagnosis of elbow fractures.
3. US compared with X-ray in the diagnosis of distal radius fractures.
4. US compared with X-ray in the diagnosis of metacarpal fractures.
5. US compared with X-ray in the diagnosis of lower limb fractures.
6. US compared with X-ray in the diagnosis of metatarsal fractures.
7. US compared with X-ray in the diagnosis of clavicle fractures.

**Figure 1:**
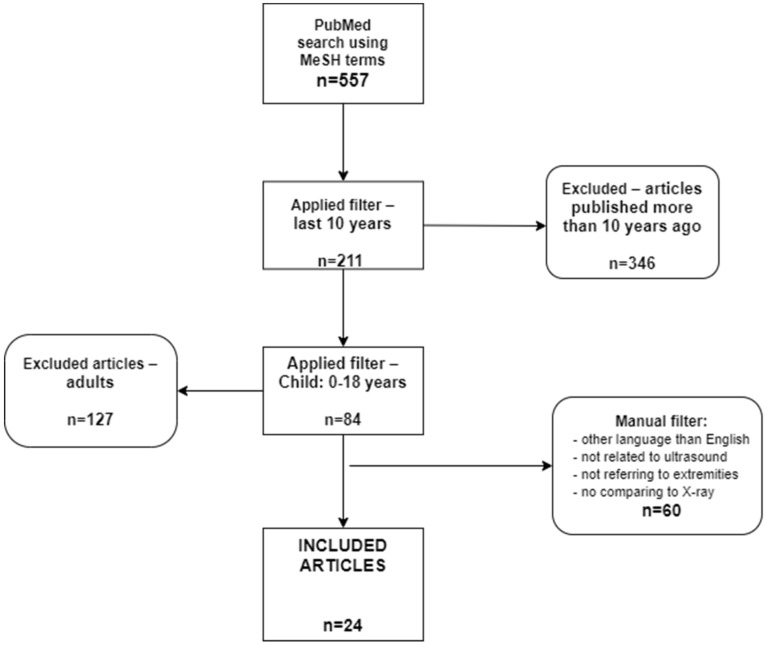
PRISMA flowchart. Literature review.

The scheme below shows the used search strategy and the applied filters (Figure 1).

## 3. Results

A total of 24 articles were included in the study. Their main information is found in the table below (Table 1).

From a total of 2247 patients included in the articles used for this review, only 1855 were eligible for calculating the gender distribution. Thus, 58.32% were males. Regarding the articles that included only pediatric subjects, the mean age was 8.72 years.

In 62.5% of the studies, the ultrasound examination was carried out by emergency medical physicians or by pediatric emergency doctors. The radiologists and ultrasonographers are mentioned both in 12.5% of the reviewed articles, whereas orthopedic surgeons and sports medicine doctors were referred to in 8.33% and 4.16% of the cases, respectively. Regarding the level of training, in most of the studies, the examiners received a 1 h session of theory and practice in musculoskeletal ultrasound.

As can be seen in the tables below, regarding each anatomical region, ultrasound specificity and positive predictive value are lower in the articles in which the examiners received only one hour of training compared with those in which the performing doctors had more experience.

As mentioned above, the data from most of the papers were organized into anatomical subgroups.

### 3.1. US Compared with X-ray in the Diagnosis of Humeral Fractures

According to the observational prospective study realized by A. Akinmade, I. Ikem, O. Ayoola et al. (2018) regarding the comparison between the accuracy of X-ray and US in the fractures diagnosis and follow-up, the following results were found: 50 out of 52 fractures seen on X-ray were recognized with ultrasound. The two false-negative results obtained with ultrasound were localized on the humerus metaphysis. The sensitivity and specificity of US in these cases were of 96.2%, 100% (95% C.I.—confidence interval), with a PPV (positive predictive value) of 100% and NPV (negative predictive value) of 83.3% (95% C.I.). Thus, it has been concluded that some limits exist in using ultrasound as an alternative method of metaphysis fracture diagnostic. On the other hand, echography has the property to detect the callus earlier compared with conventional radiography [33].

O. Ackermann, J. Simanowski, K. Eckert, following a study regarding US as a diagnose method for long bone fractures published in 2020, come to the following conclusions:

– In the case of distal forearm fractures, the sensitivity and specificity of the exam was 94–97% and 97–99%, respectively;
– Regarding the proximal humerus fractures, US was carried out in four longitudinal sections, with a specificity of 100% and a sensitivity of 94% compared with the X-ray;
– Referring to the screening in case of fracture suspicion, US finds its utility especially in case of small children or in case of abuse, when it is difficult for the pediatric patients to identify the sore spot; therefore, instead of irradiating the whole limb, the US could be used for focalizing on the most probable spot of fracture [31].

I. Barata, R. Spencer, A. Suppiah et al. published in 2012 a study in which 53 patients were included, being made 98 ultrasounds. The study results were: the sensitivity of the US was higher in diagnosing the diaphyseal fractures compared with the bones’ extremities, detecting all the diaphyseal fractures, and 27 out of 29 metaphyseal or epiphyseal fractures. Moreover, echography has identified all of the bone displacements that were diagnosed on the X-ray, with a specificity of 100% regarding the necessity of fracture reduction [34].

Table 1 shows the characteristics of studies regarding long bones.

### 3.2. US Compared with X-ray in the Diagnosis of Elbow Fractures (Supracondylar Fractures)

In 2018, J. Tokarski, J.R. Avner, and J. Rabiner published a study according to which out of 42 fractures, 23 could be diagnosed by only using US; moreover, the authors affirm that elbow ultrasonography combined with the clinical suspicion of fracture has a sensitivity of 100%. They conclude the fact that the required time for an ultrasound is of approximately 3 min, compared with conventional radiography which, being a more laborious method, takes 60 min. In addition, the authors affirm that, on average, the sensitivity of US is about 88%, and the specificity was 74% in diagnosing the fractures, with a PPV of 71% and a NPV of 90% (95% C.I.) [35].

According to a study published by M. Burnier, G. Buisson, A. Ricard et al., in 2016, in 13 cases, US revealed lipohemarthrosis and posterior fat pad signs, which are positive signs for fractures. In two of these cases, the diagnosis was made only by the above-mentioned signs. Additionally, in each of the 21 patients on which the fracture was excluded through US, there were no signs of fractures on the X-ray as well. They conclude that if both the radiography and ultrasound are normal, a fracture can be excluded for good. Lipohemarthrosis represents blood and lipidic material in the interior of an articulation, appearing as a hyperechoic effusion around the articulation. The posterior fat pad appears hypoechoic on the US, whereas on the X-ray is difficult to be distinguished. The ultrasound sensitivity was 100% for fat-pad sign and 92% for lipohemarthrosis (95% C.I.) [36].

In a prospective study published by K. Eckert, O. Ackermann, N. Janssen et al. in 2013, in which the accuracy of the US was followed in the diagnosis of posterior fat pad sign, as a predictor for the elbow fractures on pediatric patients, the authors found the following results: out of 79 children, 38 had elbow fractures. The sensitivity and specificity of the posterior fat pad sign diagnosed by US were 97.3% and 90.5%, respectively [37].

N. Supakul et al., in a study published in 2015 regarding the separation of the epiphysis of the distal humerus at children aged under 3 years old, affirm that, out of 16 studied patients, conventional radiography did not diagnose the lesions in 9 cases. A total of 12 out of these 16 patients were also investigated by US, which identified in all 12 cases the fracture, with no evidence of dislocation. In the end, the authors concluded the fact that this type of fracture is often missed by conventional radiography, which is why it should be confirmed through US [38].

JE Rabiner et al. conducted a study towards determining the performance of POCUS in the diagnosis of elbow fractures. The results were the following: on 43 patients aged between 0–21 years old and with a fractured diagnosed on X-ray, the average sensitivity of POCUS was 98%, whereas the specificity was 70%, with a PPV of 62% and a NPV of 98% (95% C.I.). Rabiner et al. concluded that POCUS is very sensitive for elbow fractures and using this method for the diagnostic could reduce the necessity of X-ray, being also more accessible and requiring less time than conventional radiography [39].

The characteristics of studies regarding elbow can be seen in Table 2.

### 3.3. US Compared with X-ray in the Diagnosis of Distal Radius Fractures

In a study conducted by C. Ko, M. Baird, M. Close et al., related to the accuracy of the US in diagnosing the distal radius fractures on pediatric patients, the following aspects have been found: out of 51 patients with suspicion of distal radius fracture, 35 fractures have been found by X-ray, US detecting all of them and also one more, which was later confirmed by the callus formation. Thereby, the US sensitivity in the diagnosis of fracture is 97.1%, whereas the specificity is 100% (PPV—100%, NPV 94.1%). The authors concluded the fact that US can be successfully used in detecting distal radius fractures even by medical staff with poor experience in ultrasound [40].

AP Epema, MJB Spanjier, L Ras et al., in their study published in 2019, have noted that out of 64 fractures of the distal forearm, 61 were correctly identified by using US. Thereby, US sensitivity compared with conventional radiography was 95%, whereas the specificity was on average 86%. In addition, out of 36 patients diagnosed as not having fractures, US correctly identified 31 of them, the specificity being 86%. The PPV was found to be 92% and the NPV, 91% (95% C.I.) [41].

From a study published by K. Eckert, O. Ackermann, B. Schwieger et al. in 2012, referring to US compared with radiography in the diagnosis accuracy of metaphyseal radius fractures, the following results have been found: out of 52 metaphyseal forearm fractures diagnosed by conventional radiography, US detected all of them, its specificity being 97% and its sensitivity, 96.1%, PPV—94.3% and NPV—97.9%. The patients with no fractures were correctly diagnosed by US. The authors concluded that US is a good alternative for X-ray [42].

F.M. Chaar-Alvarez, F. Warkentine, K. Cross et al. had the following results: out of 46 fractures diagnosed by X-ray, US diagnosed 44 of them and out of 55 patients that were diagnosed with no fracture by X-ray, 51 were given the same result by US. The US accuracy was 94%, the sensitivity was 96%, specificity 93%, PPV 92%, and NPV 96% (95% C.I. The authors concluded that the US could be used for the diagnosis of the distal radius fracture in children, being a less painful method than the traditional radiography [43]. Table 3 below shows the sensitivities and specificities regarding the distal radius fractures.

S. Musa and P. Wilson published in 2015 a study in which they compared conventional radiography and ultrasound in diagnosing the ulnar and distal radius fractures, but also the calf fractures, mostly fibula. Referring to the distal radius fractures, they recorded the following results: out of 24 fractures diagnosed by X-ray, US detected all of them [29].

In the table below we can find the characteristics of studies regarding distal forearm (Table 4).

### 3.4. US Compared with X-ray in Diagnosing the Metacarpal Fractures

In the study published in 2014, E. Neri, E. Barbi, I. Rabach et al. compare radiography and ultrasound from the viewpoint of sensitivity and specificity, depending on the specialist that examined the patients—a radiologist or emergency doctor. They reached the following results: out of 79 metacarpal fractures, the radiologists identified 72 of them using ultrasound, resulting in a sensitivity of 91.1% and a specificity of 97.6%. When these fractures were examined by the emergency doctors, the ultrasound sensitivity was 91.5%, and the specificity was 96.8% (Table 5). The authors concluded that the results are similar, which means that the ultrasound can be used by an emergency doctor in order to evaluate the fractures [44].

N. Kozaci, M.O. Ay, M. Kespli et al., in a study published in 2015 regarding the efficacity of POCUS in the diagnosis and management of metacarpal fractures, came to the following results: out of 66 patients included in the study, the fractures were diagnosed at 36 patients with the help of X-ray and at 37 by using POCUS. Therefore, POCUS had a sensitivity of 92% and a specificity of 87%. The authors concluded that POCUS can be successfully used by emergency doctors for diagnosing metacarpal fractures [45].

The characteristics of studies regarding metacarpal bones can be found in Table 6.

### 3.5. US Compared with X-ray in Diagnose of the Lower Limb

In the study published by S. Musa and P. Wilson in 2015, regarding fibula fractures, they concluded that US detected the distal fibula fractures at a rate of 50% [29].

N. Kozaci, M.O. Ay, M. Avci et al., through their study from 2017 referring to the comparison between POCUS and X-ray in the diagnosis of tibial and fibular fractures, had the following results: 21 tibial fractures were detected by X-ray, whereas ultrasound detected 24 fractures. The sensitivity of POCUS regarding the tibial fractures was 100%, whereas the specificity was 93%. Regarding the fibular fractures, X-ray identified 24, whereas POCUS identified 25. The sensitivity was 100% and the specificity, was 97% (Table 7). The authors concluded that POCUS is as efficient as X-ray in the diagnosis of tibia and fibula fractures in pediatric patients [46].

Takakura et al. conducted a study on avulsion fractures of the distal fibula after a lateral ankle sprain, comparing the accuracy of ultrasound with conventional radiography, finding no significant differences between these two diagnostic methods, with ultrasound’s sensitivity being 94% and specificity at 85% (PPV of 91% and NPV of 89%) [47].

The table below (Table 8) shows the characteristics of studies regarding tibia and fibula.

### 3.6. US Compared with X-ray in the Diagnosis of Metatarsal Fractures

In the study published in 2013 by M. Yesilaras, E. Aksay, O.D. Attila et al., referring to the accuracy of US in the diagnosis of the fifth metatarsal fracture, the following results have been obtained: out of 33 patients diagnosed with a fifth metatarsal fracture with X-ray, US obtained the same results in 32 of these cases; therefore, the US’s sensitivity was 97.1%, whereas the specificity was 100%. The PPV of US regarding these bone fractures was on average 100%, whereas the NPV was 98% (95% C.I.) The conclusion was that ultrasound is an efficient diagnostic method [48].

N. Kozaci, M.O. Ay, M. Avci et al. published in 2016 a study that refers to the diagnosis and management of metatarsal fractures. They examined 72 patients, of which 28 had metatarsal fractures on X-ray and 31 on ultrasound. This means that POCUS had a sensitivity of 93% and a specificity of 89% (PPV—84%, NPV—95%). Following the results, the authors concluded that POCUS can be used as a diagnostic method for metatarsal fractures, as long as for their follow-up [49].

S. Ekinci, O. Polat, M. Gunalp et al., evaluating the accuracy of US in the assessment of trauma regarding the foot and ankle bones have noted: out of 20 patients diagnosed with fracture by using conventional radiography, ultrasound detected all of them, the most frequent fractures being on the fifth metatarsal bone. Thereby, the ultrasound sensitivity in evaluating the fracture was 100%, whereas the specificity was 99.1, with a mean PPV of 95.2% and NPV of 100% [50].

In the table below we can see the characteristics of studies regarding metatarsal bones (Table 9).

### 3.7. US Compared with X-ray in the Diagnosis of Clavicle Fractures

M. Chien, B. Bulloch, P. Garcia-Fillion published in 2011 a study referring to the comparison between X-ray and US in diagnosing the clavicle fractures. They noted the following: the sensitivity of ultrasound was 89.7%, whereas the specificity was 89.5%. The PPV was 94.6% and NPV 81% (95% C.I.). Moreover, they observed that the pain score did not substantially change during the ultrasound procedure—before it was 4.7, whereas during the procedure, it was 5.2. Furthermore, the average ultrasound took 76 min, whereas the conventional radiography was about 107 min. The conclusion was that ultrasound is not associated with a higher pain score during the procedure and the time spent in the emergency room is shorter compared with the radiography [51].

From the review published by H. Willis in 2012, regarding the accuracy of ultrasound in the diagnosis of clavicle fractures in children, the following results were found: five articles have been studied, of which four have described the sensitivities and specificities seen in the table below [52]. In the following table, we present the sensitivity and specificity of ultrasonography in diagnosing clavicle fractures, as concluded by H. Willis (Table 10).

Caroselli et al. conducted a study on both children and adults, on multiple bones—clavicula, fibula, humerus, femur, face or skull bones, patella, phalanges of the hand and foot, radius, rib, sternum, tibia, and ulna. 13.86% of the patients were children. As a result, in the pediatric population, the sensitivity of the ultrasound was 91.67%, whereas the specificity was 88.89%. The PPV was 89.2% and the NPV, 91.4% [53].

As we can see in most of the studies, no matter the examined anatomical part, the medical doctors received basic training, including theory and practice and few of them had more experience with musculoskeletal ultrasound.

In a validation study made by Caroselli et al., the participants were divided into two groups, depending on the level of experience in musculoskeletal ultrasonography of the doctors that examined the patients—standard and high-skill centers. After collecting the data from all the centers, the results were the following: the sensitivity of ultrasonography was 71% in the standard centers, whereas that of the high-skill ones was 91.67%. The specificity was 82.1% in standard centers and 88.89% in high-skill hospitals (Table 11) [54].

## 4. Discussion

Upper and lower extremities fractures are frequent in the pediatric population, being a common cause of presentation at the emergency department. Regarding the long bones fracture diagnosis, in the majority of the studied articles, the specificity and sensitivity of ultrasound are around 90–100%, and with high PPVs and NPVs, which means this method could be used as a diagnostic tool in pediatric patients with suspected fracture, using X-ray only if necessary, when ultrasound does not identify any lesion. Sonography is easy to use not just by radiologists, but also by emergency doctors, and pediatric orthopedic surgeons with a basic ultrasound course, consisting of theory and practice (most of the authors mention a 1 h course). In addition to being a complementary method for diagnosing fractures, ultrasound is helpful in monitoring the callus formation, being a repeatable method.

Studies show that ultrasound is better than conventional radiography from the point of view of the stress level of children during the investigation, being also less painful. Another conclusion is that US is superior to X-rays in detecting bone callus formation three weeks after the traumatic event [31,33].

In terms of the diagnosis of elbow fractures, ultrasound can reduce the necessity of using conventional radiography by up to 23%, which decreases the radiation ratio, being beneficial, especially for children during development. As a limitation, the authors mention the fact that not all patients have gone through a follow-up X-ray. They summarize that POCUS (point-of-care ultrasound) is a good method for diagnosing elbow fractures, being easy to be used by non-radiologists. Unless in adults, the clinical evaluation of children with fracture suspicion is more complex, the pediatric patients usually being non-compliant. As limitations, the authors mentioned that they had only one specialist that used ultrasound, suggesting that the diagnosis could be subjective. In contrast to other anatomical regions, the complete sonographic exam of the elbow is more sophisticated and requires a well-prepared specialist [35,36,37].

Regarding the diagnosis of distal radius fractures through ultrasonography even though this method is more user-friendly than conventional radiography concerning cost-efficiency, the fact that it is non-irradiant and the duration, it requires experienced doctors in any kind of ultrasonography, even if they are beginners in musculoskeletal echography. As a limitation, the authors mention the fact that ultrasonography, X-ray, and also physical exam have been performed by the same doctor [40].

Even though on long bones fractures ultrasound has higher accuracy, on metacarpal bones (and short bones in general), the diagnostic is more difficult, because they have more irregular shapes. Nevertheless, ultrasonography can be successfully used as a diagnostic measure, even on short bones. In addition to those referred above, Kozaci et al. mention the fact that, unless conventional radiography, POCUS can also detect the lesions of soft tissue, nerves, tendons, and vessels, the fact that is helpful in the correct and complete treatment establishment [45,50].

Regarding the lower extremity, the calf specifically ultrasonography is a good method in diagnosing the fractures, the patients being subjected to a smaller inconvenience and less stress and also the soft tissue and muscles lesions are easier to be detected on ultrasound, these signs being suggestive for tibial fractures [29,45]. Some authors suggest that ultrasound could be used as the first-line imaging method as an alternative to conventional X-ray in the diagnosis of distal fibula fractures [47].

Concerning the metatarsal fractures, they could be diagnosed also by beginners, with no experience or courses in musculoskeletal ultrasound [48].

Even if now, the conventional radiograph can also be portable, the devices are larger and heavier than the sonograph, and their access could be limited in some areas of the hospital. On the other hand, some limitations are mentioned for the ultrasound, such as the need for training of the clinicians and their ability to detect fractures using echography [53,54].

Additionally, other reviews and meta-analyses conducted both on adults and children show that ultrasound can be an important additional tool in diagnosing elbow fractures, having high sensitivity and specificity, but cannot replace the conventional radiography, especially in complicated fractures [55,56]. Regarding the fracture site, studies suggest that on the superior limb lesions are better seen than on the inferior one, but one of the reasons could be the fact that more studies on the upper limb than on the lower ones have been taken into account [57,58].

In contradiction to most of the studies regarding the use of ultrasound in diagnosing bone fractures, one study shows that bedside ultrasound could not be used as a replacement for conventional radiography, as the sensitivity and specificity of ultrasound, as long as its positive and negative predictive values are low in comparison with X-ray [59].

## 5. Conclusions

Musculoskeletal ultrasound is an easy-to-learn method that can help with fracture diagnosis in children. It does not require intensive training and can be used by emergency doctors also. Combined with clinical suspicion of fracture, it has a sensitivity of nearly 100% compared with conventional X-ray. Although it cannot replace conventional radiography, its high accuracy makes it a great complementary tool in fracture diagnosis. The musculoskeletal ultrasound can also diagnose the accompanying lesions, such as soft tissue, vessels, nerves injuries, but it is not suitable in complicated fractures that need reduction and for deciding the type of treatment. Moreover, ultrasound could be used for the follow-up, being able to diagnose the callus earlier than radiography.

## Figures and Tables

**Table 1:** The characteristics of studies regarding long bones.

Article	No of Patients	Age of the Patients	Examined Part	Performing Physician	Level of Training
Akinmade et al. [33]	62	(0, 15) years 5.62 ± 1.61 (mean age ± standard deviation−SD)	long bones	radiologist	-
Ackermann et al. [31]	-	(0, 12] years	upper limbs	medical doctor	-
Barata et al. [34]	53	(0, 18) 10.2 ± 3.8 (mean age ± SD)	long bones	ultrasound fellows emergency medical residents	minimum 1-year training + 1 h training on fracture ultrasound

**Table 2:** The characteristics of studies regarding elbow.

Article	No of Patients	Age of the Patients	Examined Part	Performing Physician	Level of Training
Tokarski et al. [35]	100	(0.9, 19) 7.9 ± 5.1 (mean age ± SD)	elbow	fellow physician pediatric emergency doctor	1 h training on fracture ultrasound
Burnier et al. [36]	34	(0, 15) 8 (mean age)	elbow	ultrasonographer	-
Eckert et al. [37]	79	(0, 15) 6.5 (mean age)	elbow	emergency medicine physician	>100 skeletal US examination
Supakul et al. [38]	16	(0, 2.3] 8.6 (mean age)	elbow	experienced pediatric radiologist	>16 years experience
Rabiner et al. [39]	130	(0.25, 21) 7.5 (mean age)	elbow	-	1 h training on fracture ultrasound

**Table 3:** Radius fractures. US sensitivity and specificity.

	US Sensitivity	US Specificity
Ko et al. (2019) [40]	97.1%	100%
Epema et al. (2019) [41]	95%	86%
Eckert et al. (2012) [42]	96.1%	97%
Chaar-Alvarez et al. (2011) [43]	96%	93%

**Table 4:** The characteristics of studies regarding distal forearm.

Article	No of Patients	Age of the Patients	Examined Anatomical Part	Performing Physician	Level of Training
Ko et al. [40]	51	(2, 15) 9.9 (mean age)	distal forearm	primary care sports medicine attending physician/fellow	modest training (2–3 musculoskeletal US courses)
Epema et al. [41]	100	(0, 14) 9.5 ± 3.6 (mean age ± SD)	distal forearm	EM resident/EP	basic US skill and clinical experience 1–5 years + 1 h training
Eckert et al. [42]	76	(1, 14) 8.8 (mean age)	distal forearm	-	-
Chaar-Alvarez et al. [43]	108	(1, 17) 10.3 ± 4.3 (mean age ± SD)	distal forearm	pediatric emergency medicine attending physicians fellows	bedside emergency US course
Musa et al. [29]	97	30% under 16 years	distal forearm	emergency clinicians	2–3 h of training

**Table 5:** US sensitivity, specificity, PPV, and NPV in the metacarpal fractures—radiologist vs. emergency doctor.

	US Performed by A Radiologist	US Performed by An Emergency Doctor
Sensitivity	91.1%	91.5%
Specificity	97.6%	96.8%
PPV	96%	94.7%
NPV	94.6%	94.8%

**Table 6:** The characteristics of studies regarding metacarpal bones.

Article	No of Patients	Age of the Patients	Examined Anatomical Part	Performing Physician	Level of Training
Neri et al. [44]	204	(2, 17) 12 ± 3 (mean age ± SD)	metacarpal bones	radiologist and emergency doctor	one-on-one section for 2 h
Kozaci et al. [45]	66	(5, 55) 24 ± 10 (mean age ± SD)	metacarpal bones	emergency clinicians	-

**Table 7:** Sensitivity, specificity, PPV, and NPV of US in tibial and fibular fractures.

	US Sensitivity	US Specificity	PPV	NPV
Tibia fractures	100%	93%	88%	100%
Fibula fractures	100%	97%	96%	100%

**Table 8:** The characteristics of studies regarding tibia and fibula.

Article	No of Patients	Age of the Patients	Examined Anatomical Part	Performing Physician	Level of Training
Kozaci et al. [46]	62	(5, 55)	tibia and fibula	emergency physicians	1 year bone US experience 2 h training
Takakura et al. [47]	52	(6, 12) 9 (mean age)	distal fibula	orthopaedic surgeons	>3 years muskuloskeletal US experience

**Table 9:** The characteristics of studies regarding metatarsal bones.

Article	No of Patients	Age of the Patients	Examined Anatomical Part	Performing Physician	Level of Training
Yesilaras et al. [48]	84	(14, 72) 36 ± 15 (mean age ± SD)	metatarsal bones	emergency physicians	5 demonstrating tests
Kozaci et al. [49]	72	(5, 55) 33 ± 18 (mean age ± SD)	metatarsal bones	emergency physicians	standard POCUS training
Ekinci et al. [50]	131	(16, 88) 37.2 ± 15.44 (mean age ± SD)	metatarsal bones	primary physicians	muskuloskeletal US workshops and congresses

**Table 10:** Specificity and sensitivity of ultrasound in diagnosing clavicle fractures.

Authors	Year of Publishing	US Sensitivity	US Specificity
J.D. Moritz et al.	2008	97.3%	73.7%
K.P. Cross et al.	2010	93%	86%
E.R. Weinberg et al.	2010	89%	83%
M. Chien et al.	2011	89.7%	89.5%

**Table 11:** Sensitivity and specificity of ultrasound in standard vs. high-skill centers.

	Standard Centers	High-Skill Centers
Ultrasound sensitivity	71%	91.67%
Ultrasound specificity	82.1%	88.89%
